# A Case of IgA Nephropathy in a Patient With Sarcoidosis: Confirmation of Glomerular Galactose-Deficient IgA1 Deposition

**DOI:** 10.1155/crin/7366501

**Published:** 2025-01-08

**Authors:** Yoshitaka Shimizu, Daisuke Ito, Mayumi Arakawa, Yuriko Shiozaki, Yumiko Suzuki, Seigo Ito, Naro Ohashi, Yoshihide Fujigaki, Akira Shimizu, Hideo Yasuda, Taro Misaki

**Affiliations:** ^1^Division of Nephrology, Seirei Hamamatsu General Hospital, Hamamatsu, Shizuoka, Japan; ^2^Department of Internal Medicine, Japan Self-Defense Force Iruma Hospital, Iruma, Saitama, Japan; ^3^Internal Medicine 1, Hamamatsu University School of Medicine, Hamamatsu, Shizuoka, Japan; ^4^Department of Internal Medicine, Teikyo University School of Medicine, Tokyo, Japan; ^5^Department of Analytic Human Pathology, Graduate School of Medicine, Nippon Medical School, Tokyo, Japan

**Keywords:** galactose-deficient IgA1, IgA nephropathy, liver, sarcoidosis

## Abstract

A 63-year-old Japanese housewife was admitted to our hospital because of hematuria and proteinuria lasting for 3 months. At the age of 59 years, she was diagnosed with neurosarcoidosis at another hospital, and she received oral glucocorticoid therapy for 1 year. Her serum angiotensin-converting enzyme (ACE) and 1, 25-dihydroxyvitamin D levels were elevated. Computed tomography showed lymphadenopathy of the tracheal bifurcation and diffuse nodular shadow in the lungs and liver. Renal biopsy findings were compatible with IgA nephropathy without noncaseating granulomas and glomerular galactose-deficient IgA1 (Gd-IgA1) was stained in mesangial area. Because of clinical suspicion of sarcoidosis, liver biopsy was also performed, which showed inflammation with multiple noncaseating granulomas. The patient was diagnosed with IgA nephropathy coincident with sarcoidosis. After oral administration of prednisolone, mild proteinuria persisted; however, serum creatinine level was normalized, hematuria disappeared, and serum ACE and 1, 25-dihydroxyvitamin D levels returned to normal. Although some patients with sarcoidosis occasionally present with glomerulonephritis, there have been few case reports of sarcoidosis with IgA nephropathy. This was the first case report in which glomerular Gd-IgA1 was identified in a patient with IgA nephropathy and sarcoidosis.

## 1. Introduction

Sarcoidosis is a multisystemic granulomatous disease of unknown etiology [1]. It is characterized by the presence of noncaseating granulomas in several organs [1, 2]. The average incidence rate of sarcoidosis is 1.01 per 100,000 inhabitants in Japan [3]. Sarcoidosis has a variable clinical presentation and usually presents with bilateral hilar lymphadenopathy and lung infiltration, and multiple organs may be involved [4, 5]. Renal involvement in sarcoidosis is uncommon [5]. The main renal abnormalities are interstitial nephritis, or rarely, glomerulonephritis [5]. A variety of glomerular lesions have been described, including membranous nephropathy, minimal-change disease, proliferative or crescentic glomerulonephritis, focal segmental glomerulosclerosis, and IgA nephropathy [6].

IgA nephropathy is the most common type of primary glomerulonephritis worldwide, especially in Asia [7], and it is characterized by predominant or codominant IgA deposition in the mesangial area [8]. Approximately 30%–40% of patients with IgA nephropathy progress to end-stage kidney disease within 20 years [9, 10]. However, there is no disease-targeted treatment for IgA nephropathy because the pathogenesis remains unknown [8]. The pathogenesis of IgA nephropathy is thought to involve impaired immunoregulation [11] and genetic predisposition [12, 13]. Several studies have suggested that galactose-deficient IgA1 (Gd-IgA1) plays a crucial role in the pathogenesis of IgA nephropathy. A recently developed novel lectin-independent enzyme-linked immunosorbent assay (ELISA) utilizing an anti-Gd-IgA1 monoclonal antibody (KM55) was introduced [14]. Immunofluorescence studies using the KM55 antibody have demonstrated glomerular deposition of Gd-IgA1, offering new insights into the potential role of Gd-IgA1 as a crucial effector molecule in IgA nephropathy [14, 15]. Gd-IgA1 has been specifically detected in IgA nephropathy and IgA vasculitis, distinguishing it from other renal diseases [15].

Here, we present a case report describing the identification of glomerular deposition of Gd-IgA1 in a patient with IgA nephropathy and sarcoidosis.

## 2. Case Report

A 63-year-old Japanese housewife was admitted to our hospital because of hematuria and proteinuria lasting for 3 months. At the age of 59 years, she was diagnosed with neurosarcoidosis with facial nerve paralysis as assessed by enhancement of the facial nerve on MRI, bronchoalveolar lavage fluid analysis, and lymphadenopathy of the tracheal bifurcation at another hospital. Her serum creatinine level was normal (0.6 mg/dL). Urinalysis showed no proteinuria and no hematuria. Neurosarcoidosis was treated with oral prednisolone (1 mg/kg), which was gradually reduced and then discontinued at the age of 60 years. She was not followed up at that hospital thereafter.

On admission to our hospital, the patient's body temperature was 36.7°C, blood pressure 156/95 mmHg, and pulse rate 116 beats/min. There was no lymphadenopathy, rash, erythema, or abnormal lung sounds. Abdominal and eye examinations were normal. Her medical history included hypertension and dyslipidemia. She was prescribed candesartan cilexetil for hypertension. The laboratory findings are summarized in Table 1. Urinalysis showed elevated proteinuria (protein excretion, 0.7 g/day), hematuria (red blood cells 30/high-powered field), and increased urinary *α*1-microglobulin (26.7 mg/L). Serum creatinine level was mildly elevated (1.09 mg/dL). Estimated glomerular filtration rate (eGFR) was 39 mL/min. Liver function, serum calcium, and all the hematological tests were normal. Serum IgA level was markedly elevated (760 mg/dL), serum IgG level was mildly elevated (1909 mg/dL), and IgG4 level was normal (52.3 mg/dL). Serum ACE level was elevated (36.5 U/L), and 1, 25-dihydroxyvitamin D level was mildly elevated (67.4 pg/mL). Electrocardiography was normal. Contrast-enhanced chest and abdominal computed tomography showed lymphadenopathy of the tracheal bifurcation and diffuse nodular shadow in the lungs and liver, as well as splenomegaly. Gallium-67 scintigraphy showed abnormally high uptake in the liver and spleen.

**Figure 1:**
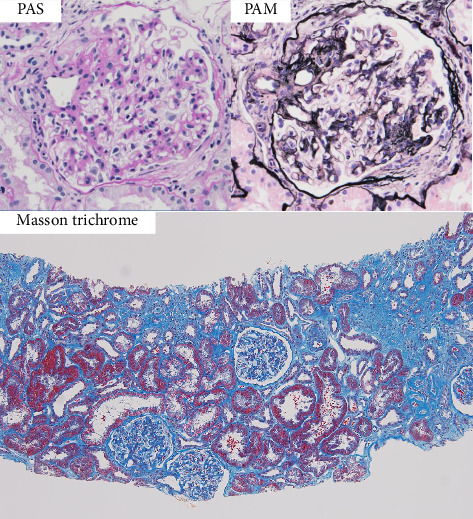
Light microscopy findings of renal biopsy. The histological pattern was mild mesangial proliferative glomerulonephritis: periodic acid-Schiff (PAS) stain, 400×; periodic acid methenarnine silver (PAM) stain, 400×. There was interstitial fibrosis and tubular atrophy but no noncaseating granulomas or tubulointerstitial nephritis (masson trichrome stain, 100×).

**Figure 2:**
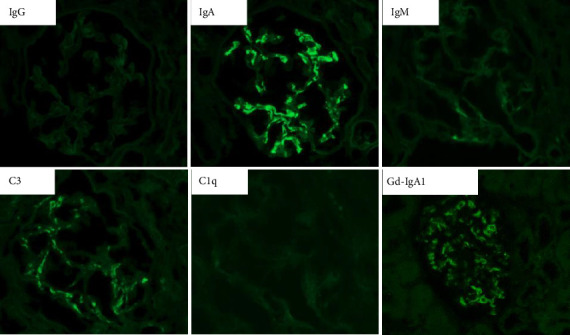
Immunofluorescence analysis of glomeruli. Immunofluorescence analysis was performed using fluorescein-isothiocyanate-conjugated antibody against IgG, IgA, Gd-IgA1, IgM, C3, and C1q. There was strong glomerular staining of IgA (2+), Gd-IgA1 (1+), and C3 (1+) in the mesangial area, while IgG, IgM and C1q were all negative.

**Figure 3:**
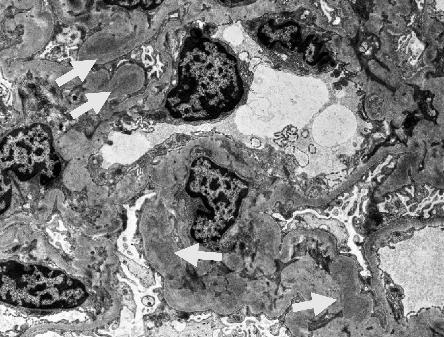
Electron microscopic analysis of glomeruli. Electron microscopy revealed electron-dense deposits (EDDs; white arrows) in the mesangial areas.

**Figure 4:**
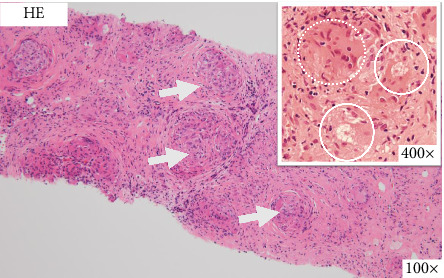
Light microscopic findings of liver biopsy. There was inflammation with multiple noncaseating granulomas (white arrows) in the liver tissue (hematoxylin and eosin [HE] stain, 100×). There were some asteroid bodies (white circle) and some multinucleated giant cells (white dot circle) in the center of the noncaseating granulomas (hematoxylin and eosin [HE] stain, 400×).

**Figure 5:**
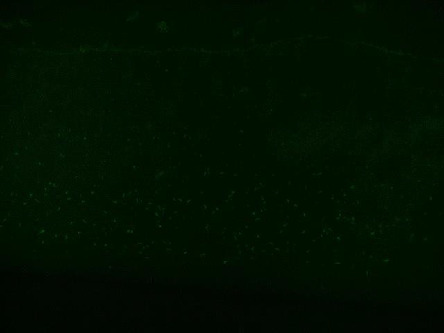
Immunofluorescence analysis of liver. Immunofluorescence analysis was performed using fluorescein-isothiocyanate-conjugated antibody against Gd-IgA1. There was no staining of Gd-IgA1.

On the basis of the above clinical findings, percutaneous renal biopsy was performed to determine the pathological characteristics of the renal dysfunction. The biopsy sample consisted of a renal cortex containing 15 glomeruli; three of which showed global sclerosis. Almost all the glomeruli showed mild mesangial proliferation (Figure 1). One glomerulus showed fibrocellular crescent formation. Although Masson trichrome staining showed 60% interstitial fibrosis and tubular atrophy, noncaseating granulomas were not seen in the biopsy sample (Figure 1). Immunofluorescence studies showed strong staining of IgA, Gd-IgA1 and complement component C3 throughout the mesangial area (Figure 2). Electron microscopy revealed electron-dense deposits in the mesangial area (Figure 3). On the basis of the renal findings, we diagnosed IgA nephropathy (Oxford Classification: M1S1E1T2C1). Liver biopsy was also performed because of clinical suspicion of sarcoidosis, which showed inflammation with multiple noncaseating granulomas (Figure 4). Immunofluorescence studies showed no staining of Gd-IgA1 in the liver (Figure 5). Thus, the patient was diagnosed with IgA nephropathy with sarcoidosis involving the liver.

**Figure 6:**
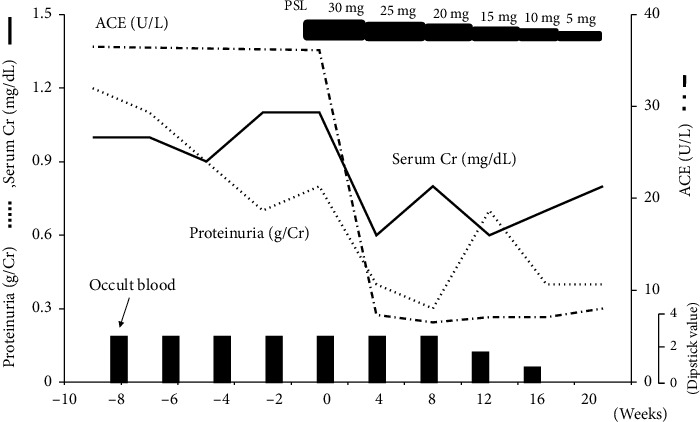
Clinical course of the patient. Alb, albumin; Cr, creatinine; ACE, serum angiotensin-converting enzyme; PSL, prednisolone. PSL, dose given in mg/day.

The patient was treated with oral prednisolone at 30 mg/day (0.6 mg/kg), which was gradually reduced to 5 mg/day over 5 months (Figure 6). After treatment, mild proteinuria persisted; however, serum creatinine level was normalized, and hematuria disappeared (Figure 6). ACE, 1, 25-dihydroxyvitamin D levels, and urinary *α*1-microglobulin also returned to normal.

## 3. Discussion

As far as we know, this is the first case report in which the glomerular deposition of Gd-IgA1 was identified in a patient with IgA nephropathy and sarcoidosis.

Although a renal biopsy was performed because of a suspicion of renal sarcoidosis, contrary to our expectations, it showed IgA nephropathy without granulomatous interstitial nephritis and noncaseating granulomas, no findings suggestive of sarcoidosis, and glomerular Gd-IgA1 was clearly stained in the mesangial area. However, liver biopsy revealed multiple noncaseating granulomas and led to diagnosis of IgA nephropathy and sarcoidosis. Sarcoidosis may have involved the liver, lungs and spleen, and caused lymphadenopathy of the tracheal bifurcation in this case. High serum levels of ACE and 1, 25-dihydroxyvitamin D also indicated the presence of active sarcoidosis.

Although our case was an accidental complication of primary IgA nephropathy and sarcoidosis, we considered there might have been an association between IgA nephropathy and sarcoidosis.

The precise pathogenesis of sarcoidosis is still unknown [5]. It has been reported that sarcoidosis is occasionally complicated with glomerulonephritis [6] and, in some cases, IgA nephropathy [6, 16–30]. Since Teilum reported the association of sarcoidosis and glomerulonephritis in 1951 [31], increased use of renal biopsy has resulted in sporadic reports of sarcoidosis and glomerulonephritis [5, 6]. There are several types of glomerulonephritis with sarcoidosis, including membranous glomerulonephritis, focal segmental glomerulonephritis, and membranoproliferative glomerulonephritis [6]. Löffler et al. reported that 26% of renal sarcoidosis cases diagnosed with renal biopsy were complicated with IgA nephropathy [32]. We searched PubMed for reports of IgA nephropathy with sarcoidosis and found 17 cases of IgA nephropathy with sarcoidosis (Table 2). These reported cases indicate that the coexistence of IgA nephropathy and sarcoidosis occasionally occurs.

It is presumed that the sarcoidosis was reactivated after the discontinuation of steroid therapy administered by the previous physician, and that IgA nephropathy in this case was complicated by an immune mechanism related to the sarcoidosis. In this case, the patient presented with sarcoidosis involving the liver, and elevated serum IgA levels were observed. Initially, we thought secondary IgA nephropathy, which was mediated by elevated serum IgA due to impaired IgA metabolism in the liver. However, in this case, strong glomerular deposition of Gd-IgA1 was detected, suggesting that primary IgA nephropathy due to immunological mechanisms was thought to have occurred. With regard to the pathogenesis of IgA nephropathy, the 4-HIT hypothesis, including production of Gd IgA1 (Hit 1), IgG or IgA autoantibodies that recognize Gd-IgA1 (Hit 2), and their subsequent immune complexes formation (Hit 3) and glomerular deposition (Hit 4), has been widely supported by many studies [33]. Gd-IgA1 is believed to play a significant role in 4-HIT hypothesis [33]. Lymphoid tissues are thought to be associated with the induction of Gd-IgA1 [34]. Additionally, it is well-established that innate immune activation through Toll-like receptor nine is linked to the production of Gd-IgA1 [35, 36]. Given the association of lymphoid tissue abnormalities with sarcoidosis and the reported activation of Toll-like receptor nine in the analysis of bronchoalveolar lavage cells and multinucleated giant cells from sarcoidosis patients [37], there may be a potential mechanism by which sarcoidosis triggers the production of Gd-IgA1. We expected Gd-IgA1 to be positive in plasma cells around multiple noncaseating granulomas of the liver; however, Gd-IgA1 was negative. We could not directly prove a relationship between sarcoidosis and Gd-IgA1 in this case. Differential diagnosis of primary IgA nephropathy from secondary IgA nephropathy is not easy, because there are no specific histological features to distinguish them. Further studies on a larger number of cases are required to confirm the association between IgA nephropathy and sarcoidosis.

Regarding the treatment of this patient, a consensus statement from sarcoidosis experts endorses glucocorticoids as the primary treatment [38, 39], and many studies have reported the effects of glucocorticoids on IgA nephropathy [40, 41]. In almost all of the cases of IgA nephropathy with sarcoidosis in Table 2, treatment with glucocorticoids was selected. In sarcoidosis, there is no definitive consensus of the glucocorticoid optimal dosage, but Kumer et al. suggest initiate glucocorticoid therapy at 20–40 mg/day [42]. In our case, we treated patient with oral prednisolone at 30 mg/day (0.6 mg/kg), glucocorticoid was effective in both IgA nephropathy and sarcoidosis.

We encountered a case of IgA nephropathy coincident with sarcoidosis in which glomerular deposition of Gd-IgA1 was identified.

## Figures and Tables

**Table 1:** Laboratory data.

Blood	Normal reference value	Patient data
White blood cells	3300–8600/μL	8570/μL
Hemoglobin	11.6–14.8 g/dL	13.9 g/dL
Platelet count	15.8–34.8 × 104/μl	27.7 × 104/μl
Creatinine	0.46–0.79 mg/dL	**1.09 mg/dL**
eGFR	> 60 mL/min	**39 mL/min**
Urea nitrogen	8–20 mg/dL	20 mg/dL
Total protein	6.6–8.1 g/dL	8.0 g/dL
Albumin	4.1–5.1 g/dL	3.5 g/dL
HbA1c	4.9%–6.0%	5.7%
Blood glucose level	73–109 mg/dL	104 mg/dL
Total cholesterol	142–248 mg/dL	107 mg/dL
Sodium	138–145 mEq/L	140 mEq/L
Potassium	3.6–4.8 mEq/L	3.8 mEq/L
Calcium	8.8–10.1 mg/dL	9.8 mg/dL
D-dimer	< 1.0 μg/mL	**6.7 μg/mL**
HBs-antigen		Negative
HBs-antibody		Negative
HBc-antibody		Negative
HCV-antibody		Negative
HIV-antibody		Negative
CRP	0.0–0.1 mg/dL	0.4 mg/dL
IgG	861–1747 mg/dL	1909 mg/dL
IgA	93–393 mg/dL	**760 mg/dL**
IgM	50–269 mg/dL	67 mg/dL
IgG4	11–121 mg/dL	52.3 mg/dL
C3	86–160 mg/dL	133 mg/dL
C4	17–45 mg/dL	31 mg/dL
CH50	32–49 mg/dL	65 mg/dL
T-SPOT		Negative
Rheumatoid factor	0–15 mg/dL	1.0 mg/dL
Antinuclear antibody	1/40 <	1/40
Anti-DNA-antibody	< 6.0 IU/mL	< 2.0 IU/mL
MPO-ANCA	< 3.5 U/mL	< 1.0 U/mL
PR-3-ANCA	< 3.5 U/mL	< 1.0 U/mL
Anti-GBM antibody	< 3.0 U/mL	< 2.0 U/mL
Serum protein-electrophoresis		Normal-pattern
ACE	8.3–21.4 U/mL	**36.5** **U/mL**
sIL-2R	145–519 U/mL	**2230** **U/mL**
1, 25-Dihydroxyvitamin D	20.0–60.0 pg/mL	**67.4** **pg/mL**
Antimitochondrial antibody m2	< 7.0 Units	< 1.5 Units

*Urinalysis*		
Proteinuria	(−)–(±)	**2+**
White blood cells	0–4/HPF	**5**–**9/HPF**
Protein	0.0–0.1 g/day	**0.7 g/day**
Occult blood	(−)–(±)	**3+**
Red blood cells	0–4/HPF	**30–40/HPF**
*α*1-microglobulin	0.5–9.5 mg/L	**26.7 mg/L**
N-acetyl-*β*-d-glucosaminidase	0.2–5.6 U/L	**16.5** **U/L**

*Note:* Bold values indicate outliers. CH50, 50% hemolytic unit of complement; HbA1c, glycosylated hemoglobin A1c; T-SPOT, tuberculosis-specific interferon *γ*.

Abbreviations: ACE, angiotensin-converting enzyme; CRP, C-reactive protein; eGFR, estimated glomerular filtration rate; GBM, glomerular basement membrane; HBc, hepatitis B core; HBs, hepatitis B surface; HCV, hepatitis C virus; HIV, human immunodeficiency virus; HPF, high-power field; MPO-ANCA, myeloperoxidase-antineutrophil cytoplasmic antibodies; PR3-ANCA, proteinase-3-antineutrophil cytoplasmic antibodies; sIL-2R, soluble-interleukin-2-receptor.

**Table 2:** Literature review of IgA nephropathy with sarcoidosis.

	Age, years	Sex	Biopsy of granuloma lesion	Treatment	Reference
Saeki et al. 1981	55	F	Unknown	Dipyridamole	[16]
Taylor, Fisher, and Hoffbrand 1982	32	M	Lymph node	—	[17]
Kishi et al. 1984	37	M	Bone marrow lung	—	[18]
Murray et al. 1987	23	M	Mediastinal lymph	PSL	[19]
Chung-Park, Lam, and Yazdy 1990	21	F	Skin lymph node	—	[20]
Anwar and Gokal 1993	42	F	Mediastinal lymph node	Spontaneous	[21]
Tateno, Kobayashi, and Kobayashi 1994	36	M	Skin	PSL	[22]
Nishiya et al. 1996	49	F	Spleen	ACE-i	[23]
Taylor and Ansell 1996	65	M	Liver	PSL	[24]
Nishiki et al. 1999	63	M	Paratracheal lymph nodes	PSL	[25]
Hamada et al. 2003	65	F	Lymph node skin	PSL	[26]
Hamada et al. 2003	62	M	Skin	Dilazep	[26]
Hamada et al. 2003	32	F	Lung	—	[26]
Khan, Hodges, and Lord 2005	41	F	Lower lung lobe	PSL	[27]
Mahfoudhi et al. 2015	38	M	Salivary gland	PSL	[28]
Akbari, Shahani, and Ranae 2019	56	F	Lung kidney	PSL	[29]
Kotwica-Strzałek et al. 2022	51	F	Mediastinal lymph node	PSL cyclophosphamide	[30]

Abbreviations: ACE-i, angiotensin-converting enzyme inhibitor; PSL, prednisolone.

## Data Availability

All data are included in the manuscript.
